# Characterization of the complete mitochondrial genome of an endemic species in China, *Aulocera merlina* (Lepidoptera: Nymphalidae: Satyrinae) and phylogenetic analysis within Satyrinae

**DOI:** 10.1002/ece3.11355

**Published:** 2024-04-30

**Authors:** Qinghui Shi, Jinling Xie, Jialing Wu, Shengchung Chen, Gang Sun, Juncheng Zhang

**Affiliations:** ^1^ Fujian Provincial Key Laboratory of Resources and Environment Monitoring & Sustainable Management and Utilization Sanming University Sanming China; ^2^ Medical Plant Exploitation and Utilization Engineering Research Center Sanming University Sanming China

**Keywords:** *Aulocera merlina*, Lepidoptera, mitochondrial genome, phylogeny, Satyrinae

## Abstract

The mitochondrial genome (mitogenome) has been extensively used as molecular markers in determining the insect phylogenetic relationships. In order to resolve the relationships among tribes and subtribes of Satyrinae at the mitochondrial genomic level, we obtained the complete mitogenome of *Aulocera merlina* (Oberthür, 1890) (Lepidoptera: Nymphalidae: Satyrinae) with a size of 15,259 bp. The mitogenome consisted of 37 typical genes, including 13 protein‐coding genes (PCGs), 2 ribosomal RNA genes (rRNAs), 22 transfer RNA genes (tRNAs), and an A + T‐rich region. The gene organization and arrangement were similar to those of all other known Satyrinae mitogenomes. All PCGs were initiated with the canonical codon pattern ATN, except for the *cox1* gene, which used an atypical CGA codon. Nine PCGs used the complete stop codon TAA, while the remaining PCGs (*cox1*, *cox2*, *nad4*, and *nad5*) were terminated with a single T nucleotide. The canonical cloverleaf secondary structures were found in all tRNAs, except for *trnS1* which lacked a dihydrouridine arm. The 448 bp A + T‐rich region was located between *rrnS* and *trnM*, and it included the motif ATAGA followed by a 19‐bp poly‐T stretch and a microsatellite‐like (TA)_6_ element preceded by the ATTTA motif. The phylogenetic tree, inferred using Bayesian inference and maximum likelihood methods, generated similar tree topologies, revealing well‐supported monophyletic groups at the tribe level and recovering the relationship ((Satyrini + Melanitini) + ((Amathusiini + Elymniini) + Zetherini)). The close relationship between Satyrina and Melanargiina within the Satyrini was widely accepted. Additionally, Lethina, Parargina, and Mycalesina were closely related and collectively formed a sister group to Coenonymphina. Moreover, *A. merlina* was closely related to *Oeneis buddha* within the Satyrina. These findings will provide valuable information for future studies aiming to elucidate the phylogenetic relationships of Satyrinae.

## Graphical abstract

In this paper, the complete mitochondrial genome sequence of an endemic species in China, *Aulocera merlina* (Lepidoptera: Nymphalidae: Satyrinae) was determined and compared with that of other known mitogenomes of Satyrinae species. Moreover, the phylogenetic trees were reconstructed based on the available mitogenome sequences, including the newly sequenced mitogenome, to gain a better understanding of the phylogenetic relationships among the major lineages of the Satyrinae.

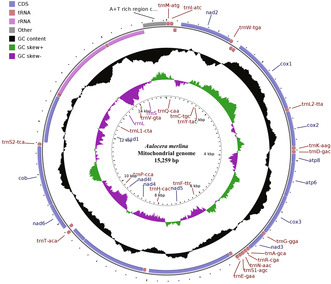

## 1 INTRODUCTION

The Satyrinae, a subgroup within the Nymphalidae family, is known for its high diversity, comprising over 2500 species found across all continents except Antarctica (Ackery et al., 1999; Peña & Wahlberg, 2008). This diversity has made Satyrinae species popular model organisms in various research fields including ecology (Schmitt & Haubrich, 2008), developmental biology (Oliver et al., 2012), and conservation biology (Slamova et al., 2013). Therefore, it is crucial to accurately identify Satyrinae species and understand their evolutionary relationships for related studies. Currently, there are nine tribes and 16 subtribes defined within the Satyrinae (Marín et al., 2011). However, the phylogenetic relationships at both the tribe and subtribe levels are still not fully resolved (e.g., Chen et al., 2020; Dan et al., 2021; Marín et al., 2011; Peña et al., 2006; Peña & Wahlberg, 2008; Sun et al., 2021; Wahlberg et al., 2009; Wu et al., 2022; Yang et al., 2020; Yang & Zhang, 2015). Given the complexity of these relationships, it is essential to utilize mitochondrial genome (mitogenome) sequences of these species for taxonomic and phylogenetic analyses.

The insect mitogenome is a circular, double‐stranded molecule that encodes a highly conserved set of 37 genes. These genes include 13 protein‐coding genes (PCGs), 22 transfer RNA genes (tRNAs), and two ribosomal RNA genes (rRNAs, *rrnL*, and *rrnS*) (Boore, 1999; Cameron, 2014). Additionally, it typically contains an A + T‐rich region that includes essential regulatory elements for replication and transcription (Taanman, 1999). Mitogenomic sequences have been widely used as molecular markers in phylogenetic analysis, comparative genomics studies, and species identification due to unique features such as strict maternal inheritance, rapid mutation rate, and limited recombination (e.g., Avise, 2009; Cameron, 2014; Liu et al., 2023; Qin et al., 2019; Tyagi et al., 2020; Yan et al., 2023). Recent studies have also shown that the entire mitogenome can provide abundant information for resolving phylogenetic relationships within Lepidoptera at various hierarchical levels (Boore, 2006; Li et al., 2021; Qin et al., 2019; Wu et al., 2014, 2022). Therefore, it is important to study more taxa and mitogenomes to gain a better understanding of the internal relationships of Satyrinae.

*Aulocera merlina* (Oberthür, 1890), an endemic species in China, belongs to the subfamily Satyrinae and is mainly distributed in Sichuan, Yunnan, and other areas (Chou, 2000). Although this species is similar in appearance to *A. padma*, it is smaller in size. However, *Aulocera* has not been included in previous studies on the phylogeny of Satyrinae using mitogenome sequences. In this study, we determined the complete mitogenome of *A. merlina* and compared it with other known mitogenomes of Satyrinae species for the first time. Additionally, we reconstructed phylogenetic trees based on the available mitogenome sequences, including the newly sequenced mitogenome, to gain insight into the phylogenetic relationships among the major lineages of the Satyrinae.

## 2 MATERIALS AND METHODS

### 2.1 Sampling and DNA extraction

The adult individuals of *A.merlina* were collected from Lijiang City, Yunnan Province, P. R. China, in July 2016. They were identified based on morphological characteristics. After collection, the samples were immediately preserved in 95% ethanol and stored at −80°C under the Voucher number SMU‐20160726. Total genomic DNA was extracted from thorax muscle tissues of a single specimen using the Rapid Animal Genomic DNA Isolation Kit (Sangon Biotech, Shanghai, China). The extracted DNA was then used for 500‐bp library construction using the NEBNext Ultra DNA Library Prep Kit for Illumina sequencing.

### 2.2 Mitogenome sequencing and assembly

Sequencing was carried out on the Illumina NovaSeq 6000 platform (BIOZERON Co., Ltd., Shanghai, China). Approximately, 13841.3 Mb of raw data from *A.merlina* were generated with 150 bp paired‐end read lengths. De novo assembly with GetOrganelle v1.6.4 (Jin et al., 2020) referencing mitogenome of closely related species *Minois dryas* (GenBank accession number: NC_046591) (Shi et al., 2019) produced contigs of mitogenome. A number of potential mitochondrion reads were extracted from the pool of Illumina reads using BLAST searches against mitogenomes of related species *M. dryas* and the GetOrganelle result. The mitochondrion Illumina reads were obtained to perform mitogenome de novo assembly using the SPAdes−3.13.0 package (Nurk et al., 2017). The GetOrganelle assembly contig was optimized by the scaffolds from SPAdes‐3.13.0 result. Finally, the assembled sequence was reordered and oriented according to the reference mitogenome, thus generating the final assembled mitochondrion genomic sequence.

### 2.3 Mitogenome annotation and analyses

The mitogenome of *A. merlina* was annotated using the online MITOS tool (http://mitos.bioinf.uni‐leipzig.de/index.py) with default parameters (Bernt et al., 2013). Protein‐coding genes (PCGs) and rRNA genes were annotated by aligning them with the homologous sequence from *M. dryas*, based on the invertebrate mitochondrial genetic code. The Tandem Repeats Finder program (http://tandem.bu.edu/trf/trf.html) was utilized to predict the tandem repeats of the control region using the default parameters (Benson, 1999). The circular mitogenomic map was generated using the CGview server (http://stothard.afns.ualberta.ca/cgview_server/) (Grant & Stothard, 2008). Secondary structures for tRNAs were manually illustrated using Adobe Illustrator 2021 based on the MITOS predictions. The nucleotide composition and relative synonymous codon usage (RSCU) values were analyzed using MEGA 11.0 software (Tamura et al., 2021). The AT and GC asymmetry were represented by the values of AT‐skew and GC‐skew, calculated using the following formulas: AT‐skew = (A − T)/(A + T) and GC‐skew = (G − C)/(G + C) (Perna & Kocher, 1995). Nucleotide diversity and the ratio of nonsynonymous substitution (Ka) to synonymous substitution (Ks) for PCGs were calculated using DNASP 6.0 (Julio et al., 2017).

### 2.4 Phylogenetic analyses

Phylogenetic analyses were conducted based on the *A. merlina* and 63 other available and complete mitogenome sequences of Satyrinae species from GenBank. Two species, *Polyura nepenthes* (Charaxinae) and *Calinaga davidis* (Calinaginae), were selected as outgroups (Table 1). The 13 PCGs and two rRNAs were first aligned individually using MEGA 11.0 software (Tamura et al., 2021) and then concatenated using DAMBE7 (Xia, 2018) for phylogenetic analyses. The best model (GTR + I + G) for concatenated sequences, determined by the corrected Akaike Information Criterion using jModeltest 2.1.10 (Darriba et al., 2012), was selected. Maximum likelihood (ML) phylogenetic analysis was performed using IQ‐TREE software (Nguyen et al., 2015). Bootstrap support (BS) values were evaluated using 1000 ultrafast bootstrap replicates (Hoang et al., 2018). Bayesian inference (BI) analysis was performed using MrBayes 3.2 (Ronquist et al., 2012). Four simultaneous Markov chains were run for 20 million generations, and trees were sampled every 100 generations. A burn‐in of 25% was applied, and the remaining samples were used to generate a consensus tree and estimate the posterior probabilities (PP). The topologies of the phylogenetic trees were visualized using FigTree v1.4.2 (Rambaut, 2014).

**TABLE 1:** List of taxa analyzed in this study together with relevant information.

Subfamily	Tribe/subtribe	Species	Size (bp)	Accession number	Reference
Satyrinae (ingroup)	Satyrini/Lethina	*Lethe chandica*	15,206	MZ501804	Yan et al. (2023)
		*Lethe verma*	15,239	NC_050916	Chen et al. (2020)
		*Lethe dura*	15,259	KF906485	Unpublished
		*Lethe uemurai*	15,272	NC_050915	Chen et al. (2020)
		*Lethe sicelis*	15,239	LC541741	Nagata et al. (2020)
		*Lethe titania*	15,257	NC_050914	Chen et al. (2020)
		*Lethe syrcis*	15,252	NC_050913	Chen et al. (2020)
		*Lethe satyrina*	15,271	NC_050912	Chen et al. (2020)
		*Lethe oculatissima*	15,243	NC_050911	Chen et al. (2020)
		*Lethe nigrifascia*	15,239	NC_050910	Chen et al. (2020)
		*Lethe marginalis*	15,229	NC_050909	Chen et al. (2020)
		*Lethe helle*	15,253	NC_050908	Chen et al. (2020)
		*Lethe hayashii*	15,246	NC_050907	Chen et al. (2020)
		*Lethe baucis*	15,251	NC_050906	Chen et al. (2020)
		*Lethe baileyi*	15,225	NC_050905	Chen et al. (2020)
		*Lethe albolineata*	15,248	NC_028507	Li et al. (2016)
		*Lethe confusa* ^a^	14,945	MT654529	Unpublished
		*Ninguta schrenckii*	15,261	KF881052	Fan et al. (2016)
		*Neope goschkevitschii*	15,286	LC541740	Nagata et al. (2020)
		*Neope pulaha*	15,209	KF590543	Wu et al. (2014)
		*Neope muirheadii*	15,217	MN242789	Yang et al. (2020)
	Satyrini/Ypthimina	*Ypthima akragas*	15,227	NC_024420	Wu et al. (2014)
		*Ypthima motschulskyi*	15,232	MN242788	Yang et al. (2020)
		*Ypthima baldus*	15,304	NC_056106	Li et al. (2020)
		*Argestina inconstans*	15,219	NC_079673	Unpublished
		*Argestina pomena*	15,226	NC_070116	Unpublished
		*Callerebia polyphemus*	15,156	NC_058609	Yan et al. (2023)
		*Callerebia suroia*	15,208	KF906483	Shi et al. (2016)
	Satyrini/Mycalesina	*Mycalesis intermedia*	15,386	MN610565	Wu et al. (2020)
		*Mycalesis mineus*	15,267	NC_025762	Tang et al. (2014)
		*Mycalesis francisca*	15,279	MN242790	Yang et al. (2020)
		*Bicyclus anynana*	16,129	OX359232	Unpublished
	Satyrini/Parargina	*Lopinga achine*	15,284	MT117843	Wu et al. (2022)
		*Pararge aegeria*	15,240	KJ547676	Teixeira da Costa (2016)
		*Lasiommata deidamia*	15,244	MG880214	Sun et al. (2021)
		*Lasiommata majuscula*	15,263	MN012997	Liu et al. (2020)
		*Lasiommata megera*	15,282	OV743337	Liu et al. (2020)
	Satyrini/Coenonymphina	*Coenonympha amaryllis*	15,125	NC_046491	Zhou, Yang, et al. (2020)
		*Coenonympha tullia* ^a^	15,316	KM592972	Unpublished
		*Triphysa phryne*	15,143	NC_024551	Zhang et al. (2016)
	Satyrini/Satyrina	***Aulocera merlina***	**15,259**	**NC_068667**	**This study**
		*Davidina armandi*	15,214	NC_028505	Unpublished
		*Hipparchia autonoe*	15,435	OK094488	Dan et al. (2021)
		*Hipparchia semele*	15,223	OW121739	Unpublished
		*Minois dryas*	15,195	NC_046591	Shi et al. (2019)
		*Oeneis urda*	15,248	NC_046889	Zhou, Liang, et al. (2020)
		*Oeneis buddha*	15,259	OK094489	Dan et al. (2021)
		*Paroeneis palaearcticus*	15,942	OK094490	Dan et al. (2021)
	Satyrini/Melanargiina	*Melanargia asiatica*	15,142	NC_024550	Huang et al. (2016)
		*Melanargia caoi*	15,469	MN012999	Liu et al. (2020)
		*Melanargia meridionalis*	15,442	NC_067761	Unpublished
		*Melanargia galathea*	15,367	OV049880	Vila et al. (2022)
	Satyrini/Maniolina	*Aphantopus hyperantus*	15,240	KM592969	Unpublished
		*Maniola jurtina*	15,258	HG995237	Lohse and Weir (2021)
	Satyrini/Erebiina	*Erebia aethiops*	15,201	OV281099	Lohse and Lohse (2022)
		*Erebia ligea*	15,196	OU785248	Lohse et al. (2022)
	Melanitini	*Melanitis phedima*	15,142	KF590538	Wu et al. (2014)
		*Melanitis leda*	15,122	JF905446	Shi et al. (2013)
	Amathusiini	*Stichophthalma louisa*	15,721	KP247523	Unpublished
		*Stichophthalma howqua* ^a^	14,020	KF990129	Shi et al. (2015)
		*Stichophthalma camadeva*	15,257	OQ942883	Unpublished
	Elymniini	*Elymnias hypermnestra*	15,167	KF906484	Shi et al. (2015)
		*Elymnias malelas*	15,161	OQ942881	Unpublished
	Zetherini	*Ethope himachala*	15,531	NC_070168	Unpublished
Charaxinae (outgroup)	Charaxini	*Polyura nepenthes*	15,333	NC_026073	Shi et al. (2015)
Calinaginae (outgroup)	–	*Calinaga davidis*	15,267	NC_015480	Xia et al. (2011)

*Note:* The specie with newly sequenced mitogenome was emphasized in bold.

^a^ The mitochondrial genome of the indicated species is incomplete.

## 3 RESULTS

### 3.1 Mitogenome organization

**FIGURE 1:**
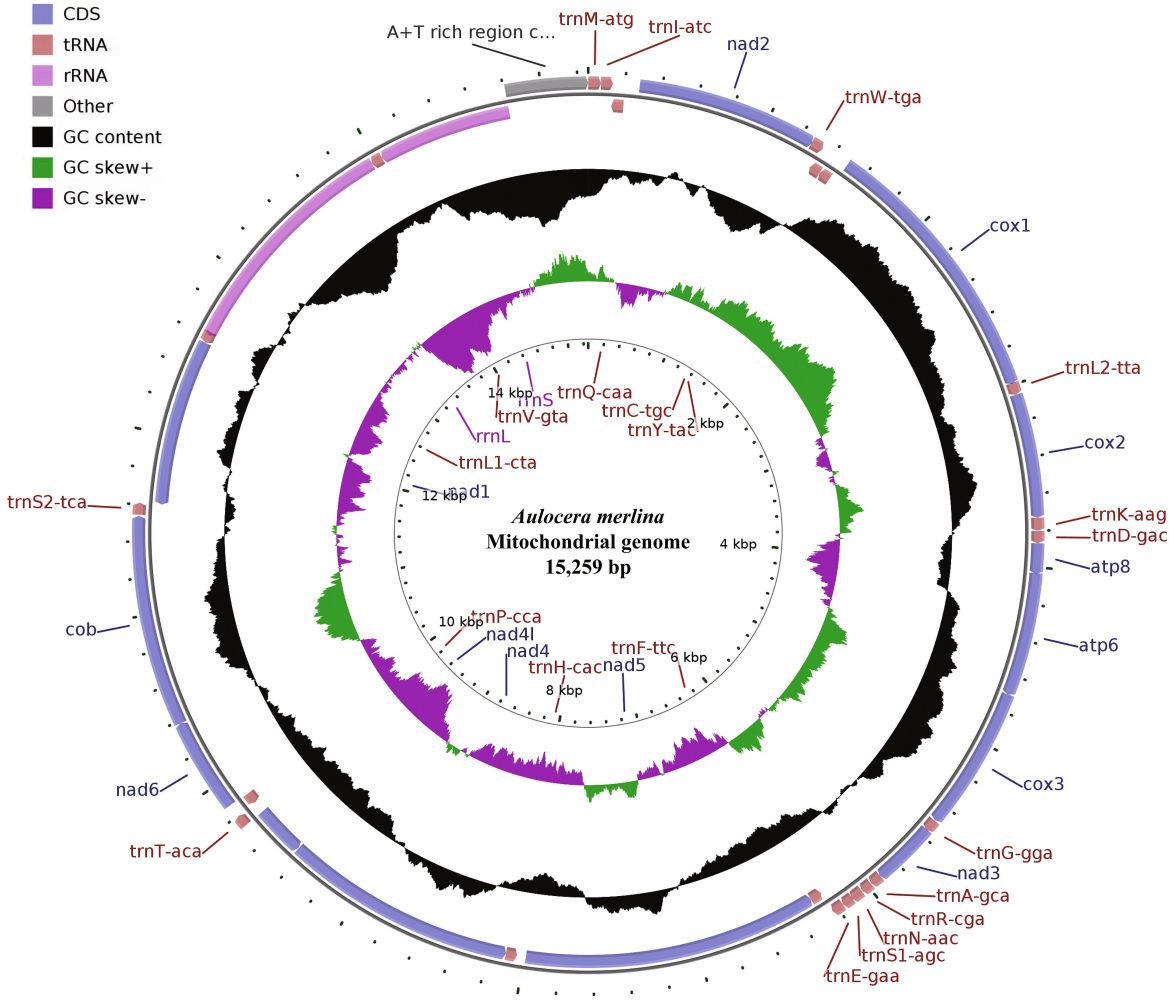
Circular maps of the *Aulocera merlina* mitogenome. Gene names on the outside of the circle indicated that these genes are located on the majority strand, whereas the others are located on the minority strand.

The complete mitogenome of *A. merlina* was a typical circular DNA molecule of 15,259 bp in size (GenBank accession number: NC_068667). It consisted of 37 genes, including 13 PCGs, 22 tRNAs, and 2 rRNAs, and an A + T‐rich region (Figure 1, Table 2). The gene order and arrangement of the newly determined mitogenome were similar to those of other butterflies. Among the genes, 23 genes (9 PCGs and 14 tRNAs) were located on the majority strand (J‐strand), while the remaining 14 genes (4 PCGs, 8 tRNAs, and 2 rRNAs) were transcribed on the minority strand (N‐strand). The nucleotide composition of *A. merlina* was significantly biased toward A + T, with a value of 79.9%. The nucleotide skewness statistics for the mitogenome showed slight T skews (−0.031) and moderate C skews (−0.224) (Table 3). The *A. merlina* mitogenome contained a total of 12 intergenic spacers, ranging in size from 1 to 53 bp, with a total length of 84 bp. The longest intergenic spacer region was located between the genes of *nad2* and *trnW*. Additionally, there were 10 overlapping regions (29 bp in total) in the mitochondrion, ranging in length from 1 to 8 bp. The longest overlapping region was found between the *trnC* and *trnY* genes (Table 2).

**TABLE 2:** Characteristics of the *Aulocera merlina* mitogenome.

Gene	Coding strand	Location	Size (bp)	Anticodon	Intergenic nucleotides	Start codon	Stop codon
*trnM*	J	1–69	69	CAU	–	–	–
*trnI*	J	70–133	64	GAU	0	–	–
*trnQ*	N	131–199	69	UUG	−3	–	–
*nad2*	J	253–1266	1014	–	53	ATT	TAA
*trnW*	J	1265–1331	67	UCA	−2	–	–
*trnC*	N	1324–1387	64	GCA	−8	–	–
*trnY*	N	1388–1452	65	GUA	0	–	–
*cox1*	J	1458–2988	1531	–	5	CGA	T
*trnL2 (UUR)*	J	2989–3055	67	UAA	0	–	–
*cox2*	J	3056–3731	676	–	0	ATG	T
*trnK*	J	3732–3802	71	CUU	0	–	–
*trnD*	J	3804–3869	66	GUC	1	–	–
*atp8*	J	3870–4034	165	–	0	ATC	TAA
*atp6*	J	4028–4705	678	–	−7	ATG	TAA
*cox3*	J	4705–5493	789	–	−1	ATG	TAA
*trnG*	J	5496–5562	67	UCC	2	–	–
*nad3*	J	5563–5916	354	–	0	ATT	TAA
*trnA*	J	5916–5979	64	UGC	−1	–	–
*trnR*	J	5981–6042	62	UCG	1	–	–
*trnN*	J	6045–6111	67	GUU	2	–	–
*trnS1 (AGN)*	J	6109–6168	60	GCU	−3	–	–
*trnE*	J	6170–6235	66	UUC	1	–	–
*trnF*	N	6236–6301	66	GAA	0	–	–
*nad5*	N	6302–8036	1735	–	0	ATT	T
*trnH*	N	8037–8100	64	GUG	0	–	–
*nad4*	N	8101–9439	1339	–	0	ATG	T
*nad4l*	N	9439–9726	288	–	−1	ATA	TAA
*trnT*	J	9732–9795	64	GUG	5	–	–
*trnP*	N	9796–9860	65	UGG	0	–	–
*nad6*	J	9863–10,387	525	–	2	ATT	TAA
*cob*	J	10,387–11,538	1152	–	−1	ATG	TAA
*trnS2 (UCN)*	J	11,545–11,609	65	UGA	6	–	–
*nad1*	N	11,608–12,564	957	–	−2	ATG	TAA
*trnL1 (CUN)*	N	12,566–12,632	67	UAG	1	–	–
*rrnL*	N	12,638–13,972	1335	–	5	–	–
*trnV*	N	13,973–14,037	65	UAC	0	–	–
*rrnS*	N	14,038–14,811	774	–	0	–	–
A + T‐rich region		14,812–15,259	448	–	0		

*Note*: Strand of the genes is presented as J for majority and N for minority strand. In the column for intergenic length, a positive sign indicates the interval in base pairs between genes, while the negative sign indicates overlapping base pairs between genes.

**TABLE 3:** Nucleotide composition and skewness of the *Aulocera merlina* mitogenome.

Feature	Size (bp)	Nucleotide frequency (%)						AT‐Skew	GC‐Skew
		A	T	G	C	A + T	G + C		
Whole genome	15,259	38.7	41.2	7.8	12.3	79.9	20.1	−0.031	−0.224
Protein‐coding genes	11,203	32.7	45.7	11.0	10.7	78.4	21.7	−0.166	0.014
1st codon position	3724	36.2	36.8	16.2	10.8	73.0	27.0	−0.008	0.200
2nd codon position	3724	21.6	48.7	13.5	16.2	70.3	29.7	−0.385	−0.091
3rd codon position	3724	40.0	51.5	3.3	5.2	91.5	8.5	−0.126	−0.224
tRNA genes	1444	41.1	39.5	11.3	8.0	80.6	19.3	0.020	0.171
rRNA genes	2109	46.2	38.7	10.1	5.0	84.9	15.1	0.088	0.338
A + T‐rich region	448	45.1	46.9	4.0	4.0	92.0	8.0	−0.020	0

### 3.2 Protein‐coding genes and codon usage

**FIGURE 2:**
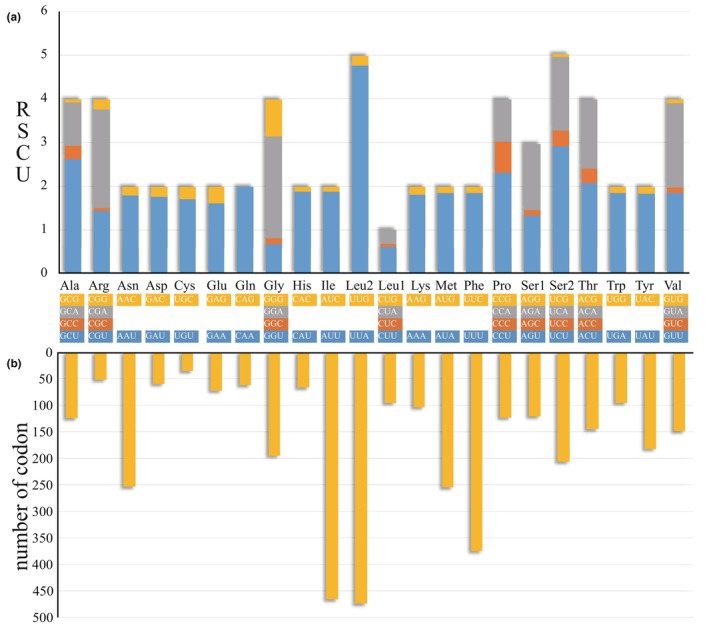
Codon usage of the *Aulocera merlina* mitogenome. Codon families are provided on the X‐axis. (a) Relative synonymous codon usage (RSCU). (b) Codon distribution.

The length of the 13 PCGs in the *A. merlina* mitogenome ranged from 165 bp for *atp8* to 1735 bp for *nad5*, totaling 11,203 bp. Among these PCGs, nine genes (*cox1*, *cox2*, *cox3*, *atp6*, *atp8*, *nad2*, *nad3*, *nad6*, and *cob*) were encoded by the majority strand, while the remaining four genes (*nad1*, *nad4*, *nad4l*, and *nad5*) were encoded by the minority strand (Figure 1, Table 2). All PCGs were initiated with the conventional ATN codons, except for *cox1* which started with the atypical CGA codon. Nine out of the 13 PCGs ended with the typical stop codon (TAA), while the remaining PCGs (*cox1*, *cox2*, *nad4*, and *nad5*) concluded with an incomplete termination codon (T‐‐). The 13 PCGs also exhibited a significant bias toward A + T content (78.4%). Notably, the third codon positions had a considerably higher A + T content (91.5%) compared to the first (73.0%) and second positions (70.3%) (Table 3). The mitogenome of *A. merlina* encodes 3724 amino acids (excluding stop codons). The relative synonymous codon usage (RSCU) values for the 13 PCGs indicated that the five most frequently used codons in the mitogenome of *A. merlina* were UUA (L), UCU (S), GCU (A), CCU (P), and GGA (G) (Figure 2a). Leucine (L, Leu) was the most commonly encoded amino acid (12.7%), followed by isoleucine (I, Ile, 12.5%), phenylalanine (F, Phe, 10.1%), methionine (M, Met, 6.9%), and asparagine (N, Asn, 6.8%). On the other hand, cysteine (C, Cys) was the least common amino acid (1.0%) (Figure 2b).

**FIGURE 3:**
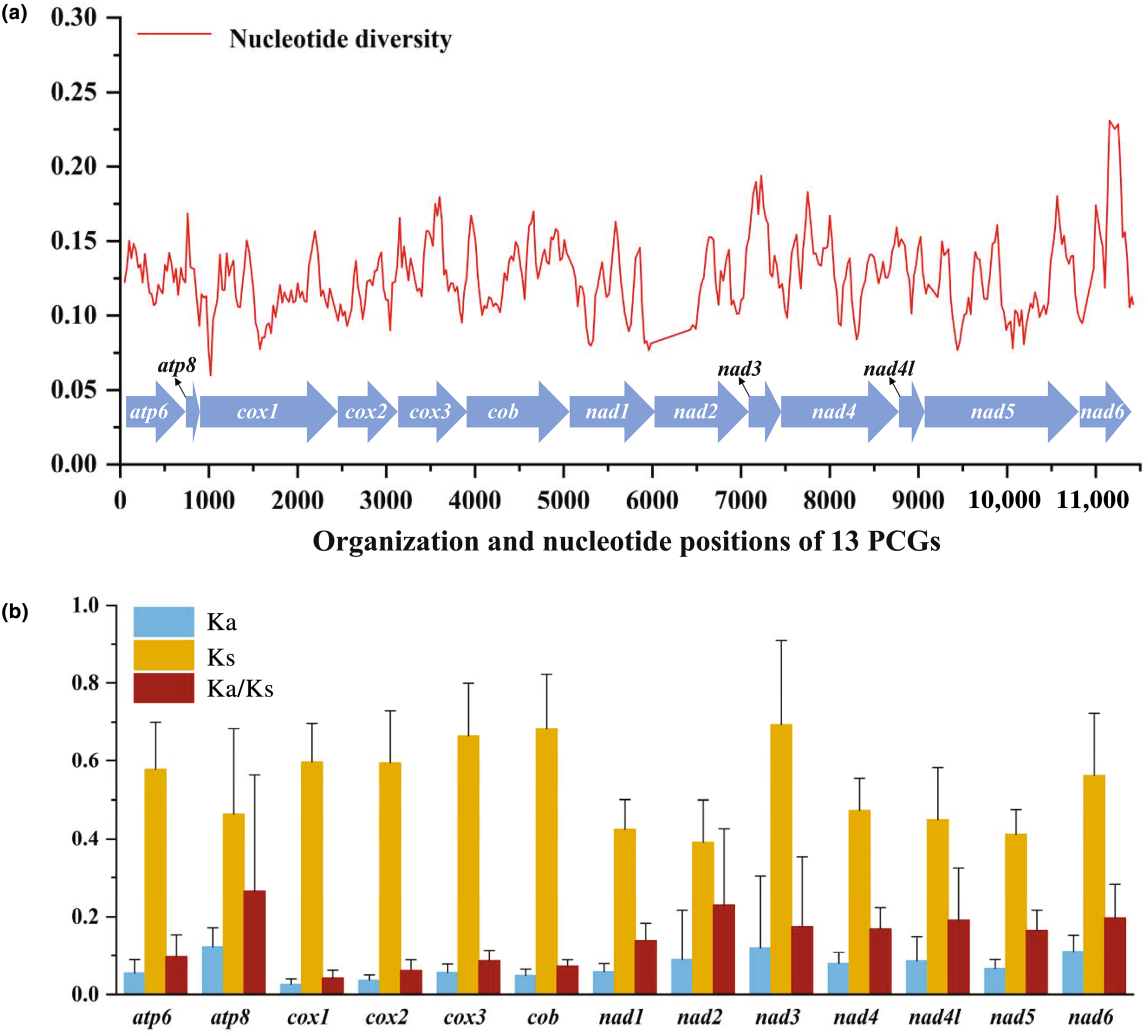
Gene variation of 13 PCGs in Satyrinae. (a) The sliding window analysis shows the value of nucleotide diversity. (b) The Ka, Ks, and Ka/Ks of each PCG among Satyrinae representatives. Ka‐nonsynonymous substitution; Ks‐synonymous substitution.

To assess the variation patterns of the 13 PCGs of Satyrinae, nucleotide diversity was calculated through sliding window analysis for each PCG. The results revealed that *cox2* was the most conserved (Pi = 0.103), while *nad3* exhibited the highest variability (Pi = 0.162) (Figure 3a). The gene with the next highest nucleotide diversity after *nad3* was *nad6* (Pi = 0.159), followed by *atp8* (Pi = 0.144), *cox3* (Pi = 0.136), *cob* (Pi = 0.133), and *nad4* (Pi = 0.132). Additionally, we calculated the values of Ka, Ks, and Ka/Ks for the 13 PCGs from 64 Satyrinae species. The results (Figure 3b) indicated that the Ka/Ks ratios were low and ranged from 0.043 (*cox1*) to 0.266 (*atp8*), suggesting that these genes underwent purifying selection (Meiklejohn et al., 2007). Consequently, the 13 PCGs are suitable for investigating phylogenetic relationships within the Satyrinae.

### 3.3 Transfer and ribosomal RNA genes

**FIGURE 4:**
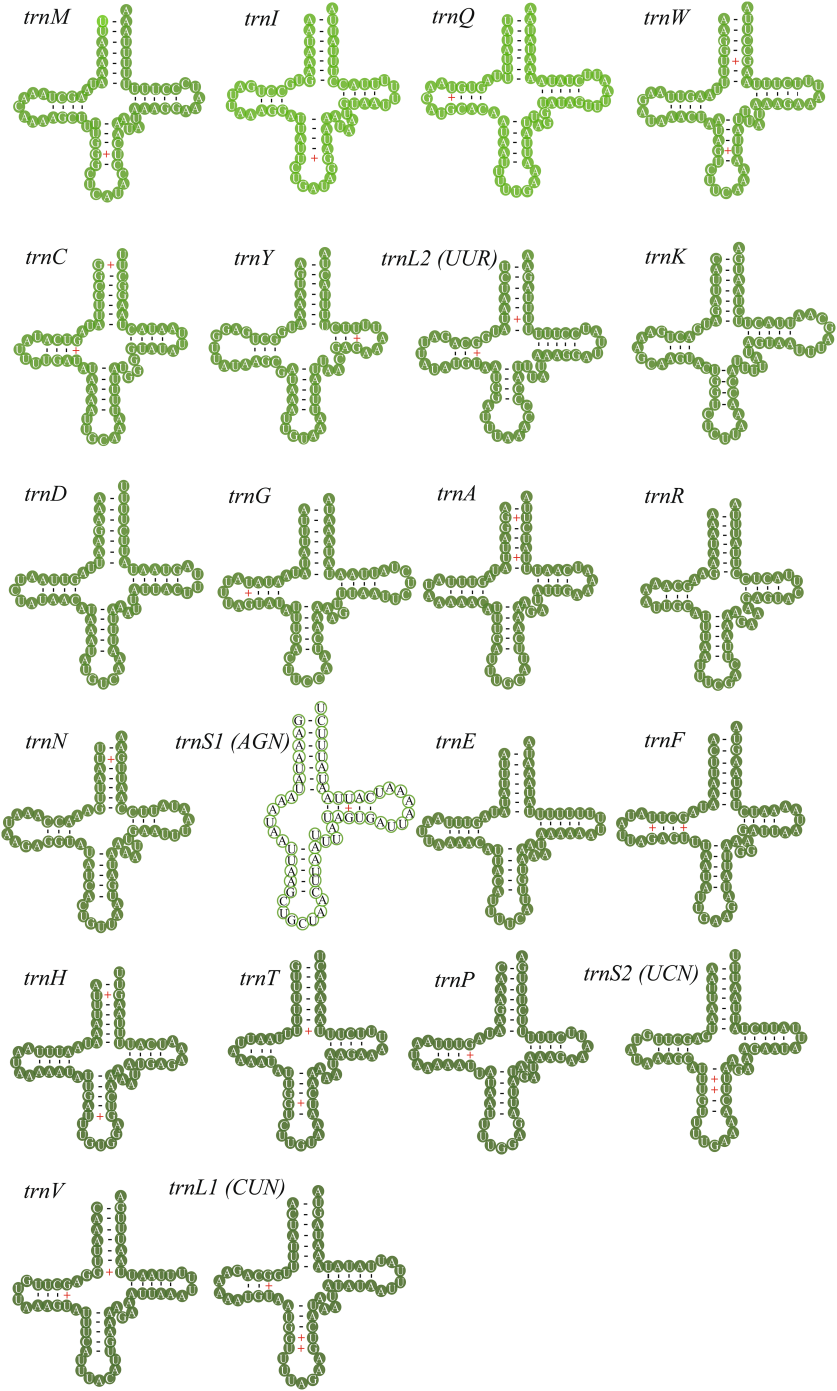
Predicted secondary cloverleaf structures for the tRNAs of the *Aulocera merlina*.

The *A. merlina* mitogenome was found to contain 22 tRNA genes, ranging in size from 60 bp (*trnS(AGN)*) to 71 bp (*trnK*) (Table 2). Out of the 22 tRNA genes, 14 were located on the majority strand (*trnA*, *trnE*, *trnD*, *trnG*, *trnK*, *trnI*, *trnL2*, *trnM*, *trnN*, *trnR*, *trnS1*, *trnS2*, *trnT*, and *trnW*), while the remaining eight were embedded in the minority strand (*trnQ*, *trnC*, *trnY*, *trnF*, *trnH*, *trnP*, *trnL1*, and *trnV*) (Figure 1, Table 2). The total length of the 22 tRNAs was 1444 bp, and the A + T content was 80.6% with positive AT‐skew (0.020) and GC‐skew (0.171). All tRNA genes exhibited a typical cloverleaf secondary structure, except for *trnS1*, which lacks the dihydrouridine (DHU) stem arm (Figure 4). The *A. merlina* mitogenome also contained two rRNA genes, both of which were embedded in the minority strand. The larger ribosomal RNA (*rrnL*) was found between *trnL1* and *trnV*, while the smaller ribosomal RNA (*rrnS*) was located between *trnV* and the A + T‐rich region (Figure 1, Table 2). The lengths of *rrnL* and *rrnS* were 1335 bp (A + T content 84.7%) and 774 bp (A + T content 85.2%), respectively (Table 3).

### 3.4 A + T‐rich region

**FIGURE 5:**
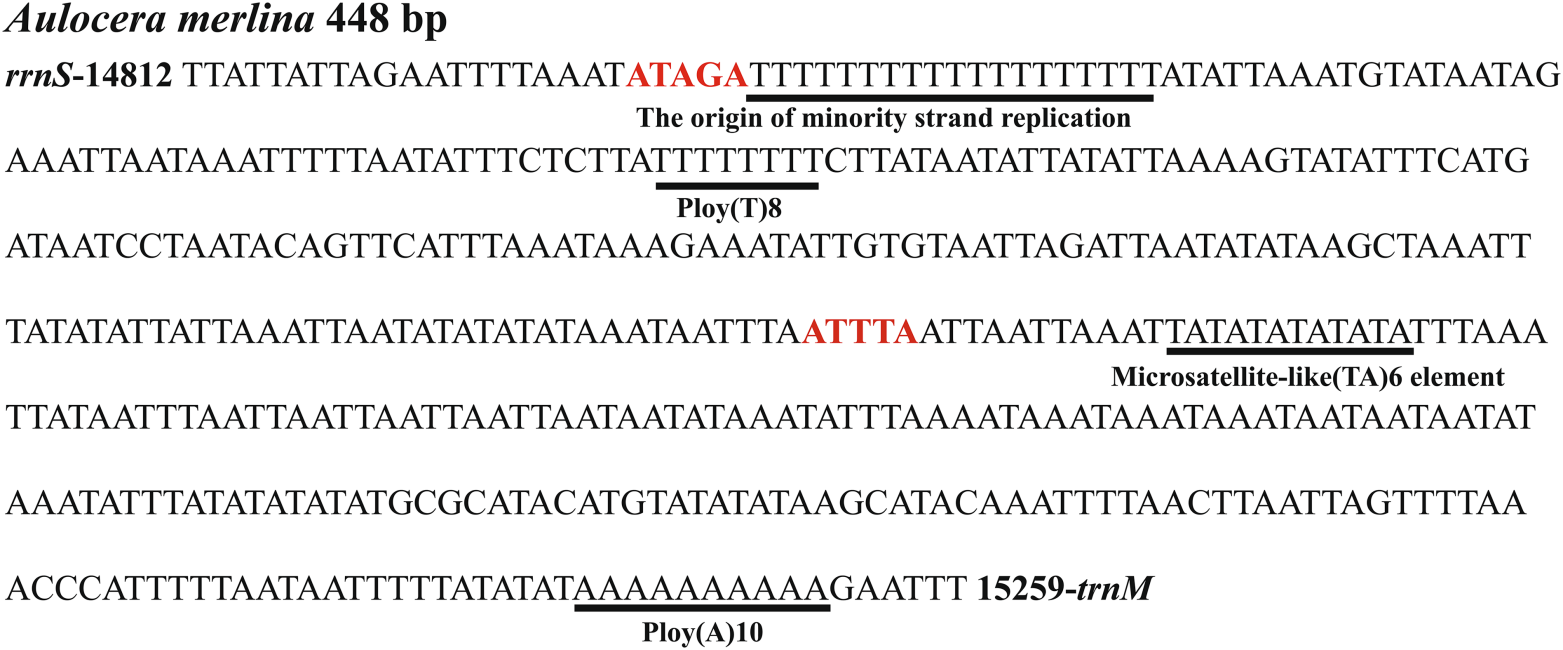
The features of A + T‐rich region in the *Aulocera merlina* mitogenome.

The A + T‐rich region of the *A. merlina* mitogenome spanned 448 bp and was situated between *rrnS* and *trnM* (Figure 1, Table 2). This region had the highest A + T content (92.0%) and a negative AT‐skew (−0.020) (Table 3), while the GC‐skew value was zero, indicating an equal proportion of G and C bases. The A + T‐rich region also contained conserved structures commonly found in lepidopteran mitogenomes, such as the ‘ATAGA’ motif followed by a 19 bp poly‐T stretch and a microsatellite‐like (TA)_6_ element preceded by the ‘ATTTA’ motif (Figure 5).

### 3.5 Phylogenetic relationships

**FIGURE 6:**
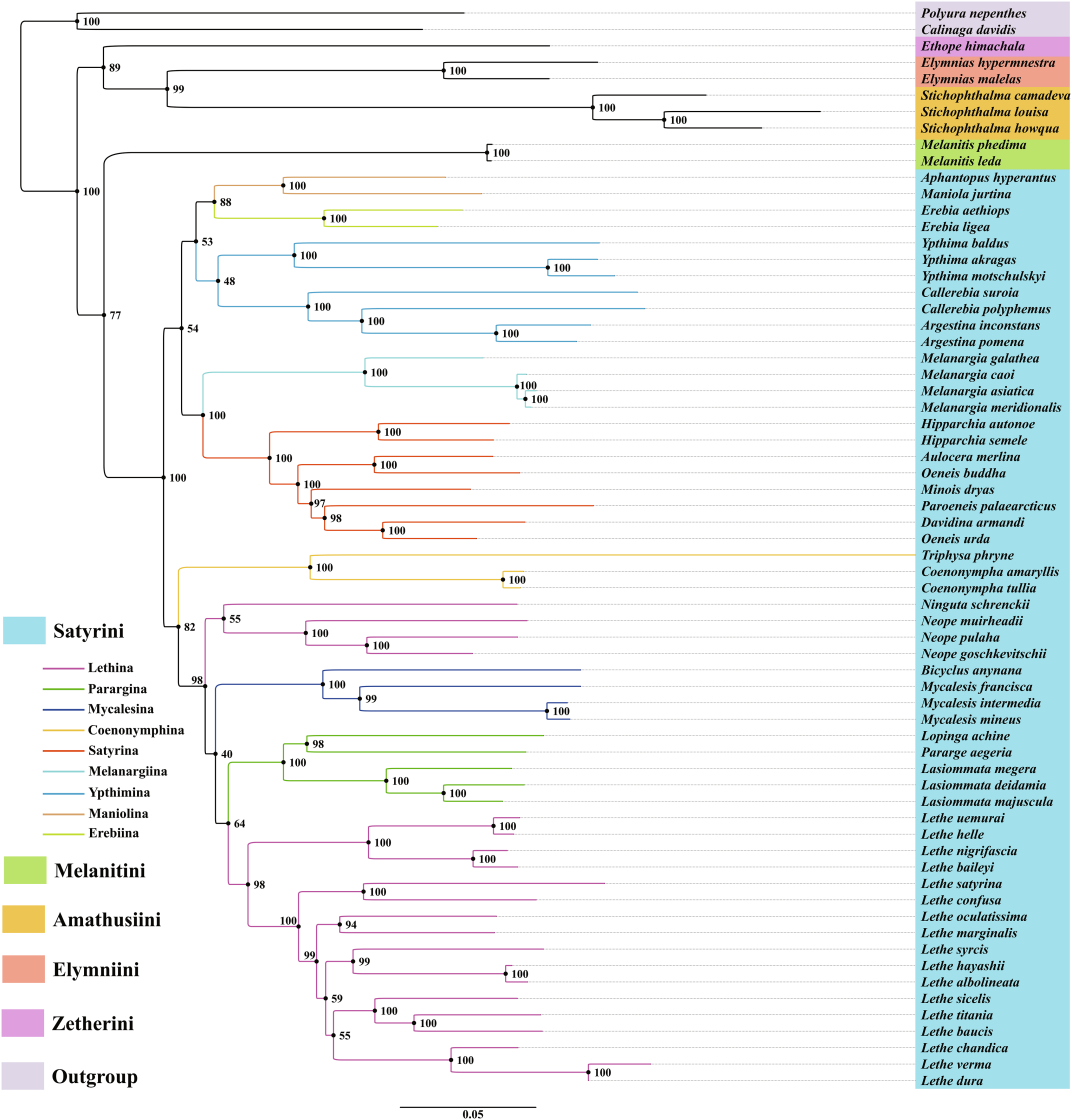
Phylogenetic tree inferred by Maximum likelihood method based on a concatenated matrix of 13 PCGs and 2 rRNAs. Bootstrap support values (BS) are shown at relevant branches.

**FIGURE 7:**
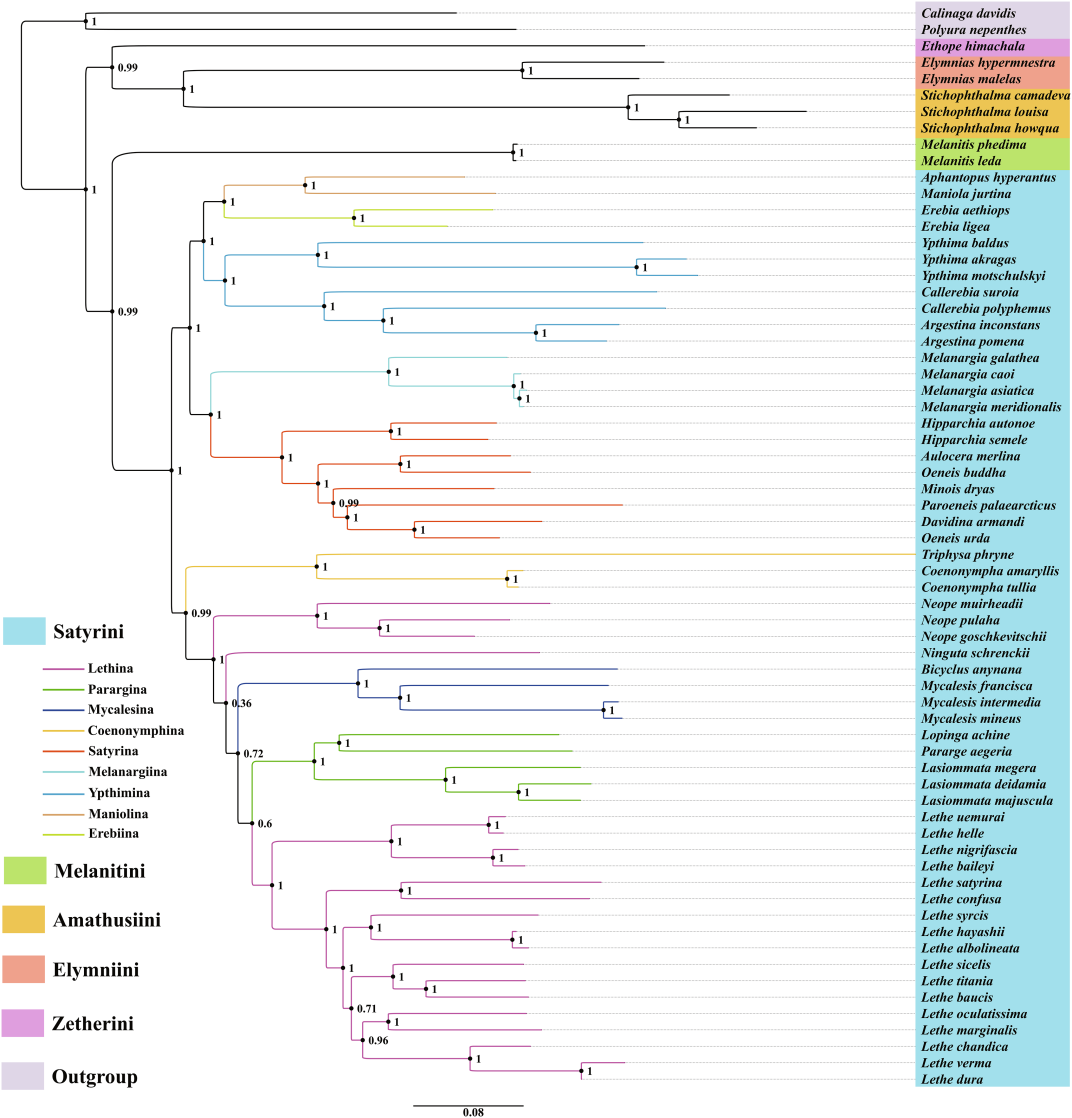
Phylogenetic tree inferred by Bayesian inference method based on a concatenated matrix of 13 PCGs and 2 rRNAs. Bayesian posterior probabilities (PP) are shown at relevant branches.

Phylogenetic analyses were conducted using concatenated nucleotide sequences of 13 PCGs and 2 rRNAs obtained from 64 known Satyrinae mitogenomes. *P. nepenthes* (Charaxinae) and *C. davidis* (Calinaginae) were used as outgroups (Table 1). Both the ML and BI methods yielded identical topologies in terms of tribal‐level relationships (Figures 6 and 7). The monophyly of Satyrini was well supported in both ML and BI analyses (BS = 100%, PP = 1.00). Moreover, Satyrini clustered with Melanitini as a sister group with strong support in the BI tree (PP = 0.96), but medium support in the ML tree (BS = 73%). Additionally, Amathusiini was identified as the sister group to Elymniini with a strong node support value (BS = 99%, PP = 1.00). The phylogenetic relationships among the five tribes of Satyrinae were found to be the same and arranged as follows: ((Satyrini + Melanitini) + ((Amathusiini + Elymniini) + Zetherini)). Within the Satyrini, except for Lethina, the subtribes Ypthima, Mycalesina, Parargina, Coenonymphina, Satyrina, Melanargiina, Maniolina, and Erebiina were monophyletic with strong support in our analyses. The relationships among the nine subtribes of Satyrini obtained from the ML and BI analyses were highly similar, although some differences exist. The BI tree showed that two well‐supported phylogenetic clades were identified from the nine sampled subtribes: clade I including Parargina, Mycalesina, Lethina, and Coenonymphina (PP = 0.99), and the remaining five subtribes constituting clade II (PP = 1.00). Furthermore, Ypthimina formed a monophyletic group with strong support (PP = 1.00) within clade II, and the phylogenetic relationships among the five subtribes recovered herein were ((Ypthimina + (Maniolina + Erebiina)) + (Satyrina + Melanargiina)) with strong support in the BI tree. The ML analysis indicated that the nine sampled subtribes of the Satyrini were also split into two clades as detected in our BI tree. However, most of the subtribe‐level relationships had weak support in the ML tree. The monophyly of the genus *Lethe* was well supported and was separated from Lethina in both ML and BI trees (BS = 98%, PP = 1.00). Furthermore, the ML analysis suggested that *Ninguta schrenckii* was clustered with three species of the genus *Neope* within Lethina, although with weak support (BS = 55%). However, in the BI analysis, *N. schrenckii* was positioned as the sister group to the grouping of the Parargina, Mycalesina, and the genus *Lethe*, but with weak support (PP = 0.36). Interestingly, both our ML and BI analyses indicated a close relationship between *A. merlina* and *Oeneis buddha* within the subtribe Satyrina, which was sister to Melanargiina with high values (BS = 100%, PP = 1.00).

## 4 DISCUSSION

**FIGURE 8:**
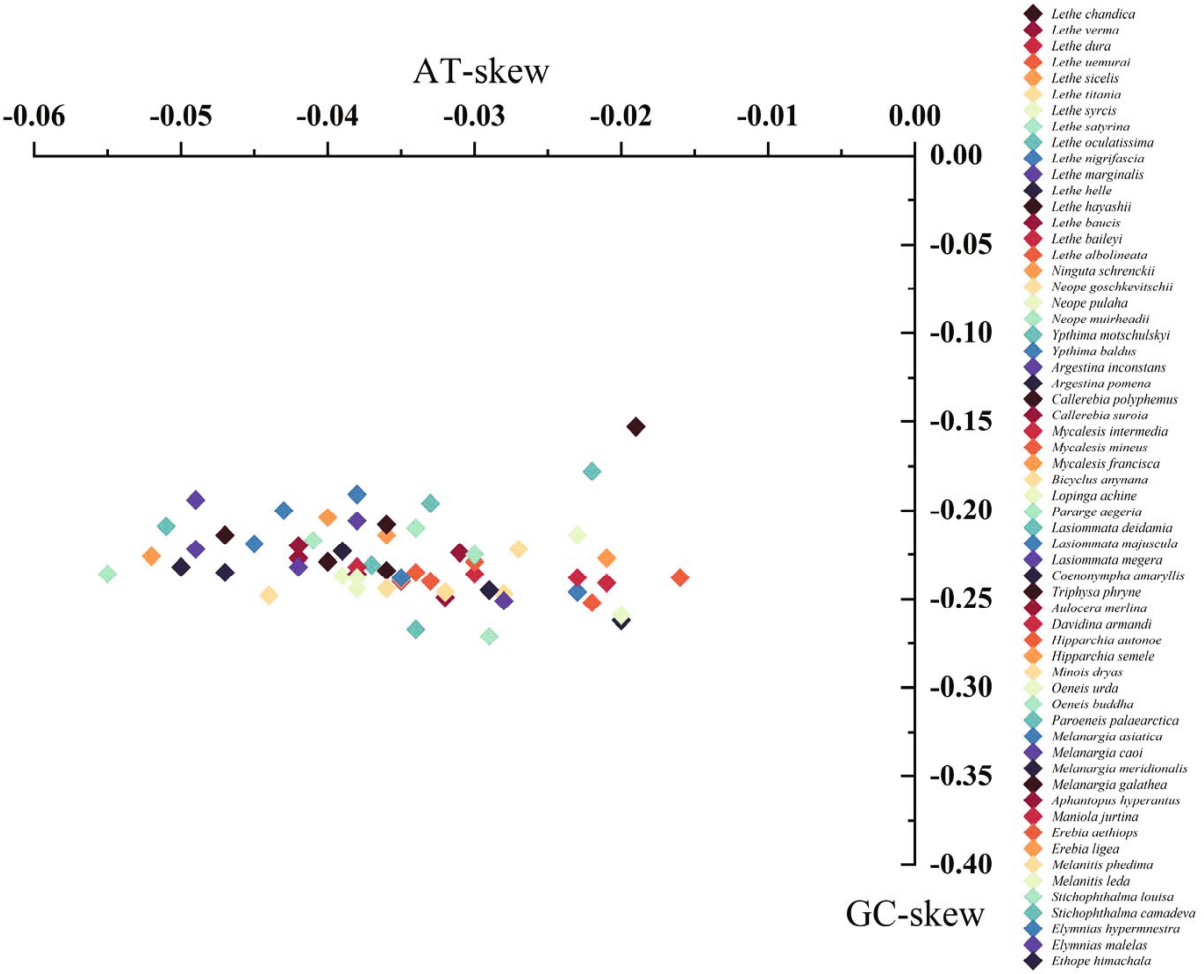
Scatter plot of AT and GC skews in the Satyrinae species.

In this study, we reported the complete mitogenome of *A. merlina* for the first time. The newly sequenced mitogenome contained 37 genes and an A + T‐rich region, with the same gene content and arrangement as other published Satyrinae mitogenomes (e.g., Chen et al., 2020; Sun et al., 2021; Yang et al., 2020). The length of the *A. merlina* mitogenome (15,259 bp) was equal to that of *Lethe dura* and *O. buddha* (Dan et al., 2021). Meanwhile, this value fell within the range of the size for other Satyrinae mitogenomes, from 15,122 bp of *Melanitis leda* (Shi et al., 2013) to 16,129 bp of *Bicyclus anynana*. The nucleotide composition of the *A. merlina* mitogenome was rich in A + T (79.9%), with slightly negative AT‐skew (−0.031) and moderately negative GC‐skew (−0.224). Similar trends have been observed in other Satyrinae species, with AT‐skew ranging from −0.055 in *Neope muirheadii* (Yang et al., 2020) to −0.016 in *Hipparchia autonoe* (Dan et al., 2021) and the GC‐skew ranging from −0.271 in *Stichophthalma louisa* to −0.153 in *Callerebia polyphemus* (Figure 8). Overall, negative AT skews and GC skews are common in Satyrinae. Furthermore, we observed a 7 bp overlap (ATGATAA) between *atp8* and *atp6*, which has been commonly found in other Lepidoptera species (Liu et al., 2018; Lu et al., 2018; Zhu et al., 2013). All PCGs were initiated by the typical ATN codon, except for *cox1*, which started with the unusual CGA codon, as seen in most other Satyrinae mitogenomes (Shi et al., 2019; Yang et al., 2020). Nine PCGs used TAA as the termination codon, while four PCGs (*cox1*, *cox2*, *nad5*, and *nad4*) terminated with an incomplete T codon. Incomplete termination codons of PCGs are commonly observed in Lepidoptera mitogenomes (Chen et al., 2020; Liu et al., 2018, 2023) and are converted into TAA by post‐transcriptional polyadenylation (Ojala et al., 1981). The canonical cloverleaf secondary structures were found in all tRNAs, except in *trnS*1, which lacked the DHU arm, as observed in other reported nymphalids (Wu et al., 2014; Yang et al., 2020). The A + T‐rich region displayed several structural characteristics commonly found in lepidopterans, such as the ATAGA motif followed by a 19 bp poly‐T stretch, and a microsatellite‐like (TA)_6_ element preceded by the ATTTA motif (Kim et al., 2014; Salvato et al., 2008).

To better understand their evolutionary relationships, we conducted a preliminary investigation using mitogenomic data. The phylogenetic relationships were reconstructed based on the concatenated nucleotide sequences of 13 PCGs and 2 rRNAs among five tribes (Satyrini, Melanitini, Amathusiini, Elymniini, and Zetherini) and nine subtribes (Lethina, Mycalesina, Parargina, Coenonymphina, Ypthima, Satyrina, Melanargiina, Maniolina, and Erebiina) within Satyrinae.

In our ML and BI analyses, the robust phylogenetic relationships among the four tribes were described as ((Satyrini + Melanitini) + ((Amathusiini + Elymniini) + Zetherini)). The close relationship between Amathusiini and Elymniini was consistent with previous mitogenomic studies (Sun et al., 2021; Wu et al., 2022; Yang et al., 2020) and the BI analysis of Peña and Wahlberg (2008). However, some studies regarded Elymniini as closer to Melanitini (Wahlberg et al., 2009; Yang & Zhang, 2015). Therefore, the tribe‐level relationships remain undefined and require further validation with a more extensive sampling of taxa.

Satyrini is the most species‐rich tribe in the Satyrinae, comprising approximately 2200 species in 13 subtribes (Marín et al., 2011). However, numerous challenges persist in resolving the systematics of Satyrini. The monophyly of Satyrini is strongly supported in the present study. Despite the limited taxon sampling within the diverse Satyrini, the Satyrina consistently appeared as a sister group to Melanargiina with strong support, which aligns with previous studies (Dan et al., 2021; Sun et al., 2021; Yang et al., 2020). Additionally, close relationships among three subtribes (Parargina, Lethina, and Mycalesina) received robust support, especially in our BI analysis, and the same pattern also recovered in previous mitogenomic studies (Dan et al., 2021; Sun et al., 2021; Wu et al., 2022; Yang et al., 2020) and multiple‐locus investigations (Peña & Wahlberg, 2008; Wahlberg et al., 2009; Yang & Zhang, 2015). Our phylogenetic results also confirmed the closer relationship between *O. urda* and *Davidina*, as previously reported (Dan et al., 2021; Lukhtanov & Dubatolov, 2020; Usami et al., 2021). Unfortunately, Lethina was not found to be a monophyletic group, and the genus *Lethe* was classified as an independent group in our phylogenetic analyses. Correspondingly, *Neope* and *Ninguta* were not placed as sister groups to *Lethe*, which contradicts previous findings (Chen et al., 2020; Dan et al., 2021; Peña et al., 2006). Furthermore, the placement of genus *Ninguta* varied across our ML and BI trees. It is important to note that this study only analyzed nine out of the 13 subtribes, which limits our ability to fully understand the phylogeny of Satyrini. Therefore, more comprehensive studies with increased sampling of mitogenome sequences are needed to improve our understanding of the evolutionary status and phylogenetic relationships of the entire Satyrini.

## 5 CONCLUSIONS

The complete mitogenome of an endemic species in China, *A. merlina* was determined and analyzed in this study. Phylogenetic trees were reconstructed using concatenated nucleotide sequences of 13 PCGs and 2 rRNAs from *A. merlina* and 63 other known Satyrinae mitogenomes. The ML and BI methods were employed, with *P. nepenthes* (Charaxinae) and *C. davidis* (Calinaginae) serving as outgroups. The gene organization and arrangement of the newly sequenced mitogenome were similar to those of other known Satyrinae mitogenomes. Both ML and BI methods produced similar topologies, supporting well‐defined monophyletic groups at the tribe level and recovering the relationship ((Satyrini + Melanitini) + ((Amathusiini + Elymniini) + Zetherini)). The close relationship between Satyrina and Melanargiina within the Satyrini was widely accepted. Additionally, Lethina, Parargina, and Mycalesina were closely related and collectively formed a sister group to Coenonymphina. Moreover, *A. merlina* was closely related to *O. buddha* within the Satyrina. These results provide valuable information for future studies on the phylogenetic relationships of Satyrinae in future studies.

## AUTHOR CONTRIBUTIONS

**Qinghui Shi:** Conceptualization (equal); data curation (equal); funding acquisition (lead); methodology (equal); project administration (lead); software (equal); validation (equal); writing – original draft (equal); writing – review and editing (lead). **Jinling Xie:** Conceptualization (equal); data curation (equal); methodology (equal); software (equal); writing – original draft (equal). **Jialing Wu:** Conceptualization (equal); data curation (equal); methodology (equal); writing – review and editing (equal). **Shengchung Chen:** Methodology (equal); validation (equal); writing – review and editing (equal). **Gang Sun:** Methodology (equal); validation (equal); writing – review and editing (equal). **Juncheng Zhang:** Supervision (lead); validation (equal); writing – review and editing (supporting).

## FUNDING INFORMATION

Natural Science Foundation of Fujian Province, Grant/Award Number: 2021J011121; Provincial Training Program of Innovation and Entrepreneurship for Undergraduates, Grant/Award Number: S202311311075.

## CONFLICT OF INTEREST STATEMENT

The authors declare no conflict of interest.

## Data Availability

The mitogenome sequence of *Aulocera merlina* has been deposited on GenBank, and assigned accession number (NC_068667) is provided.
